# Will morphing boron-based inhibitors beat the β-lactamases?

**DOI:** 10.1016/j.cbpa.2019.03.001

**Published:** 2019-06

**Authors:** Alen Krajnc, Pauline A Lang, Tharindi D Panduwawala, Jürgen Brem, Christopher J Schofield

**Affiliations:** Department of Chemistry, University of Oxford, Chemistry Research Laboratory, 12 Mansfield Road, Oxford, OX1 3TA, United Kingdom

## Abstract

The β-lactams remain the most important antibacterials, but their use is increasingly compromised by resistance, importantly by β-lactamases. Although β-lactam and non-β-lactam inhibitors forming stable acyl–enzyme complexes with nucleophilic serine β-lactamases (SBLs) are widely used, these are increasingly susceptible to evolved SBLs and do not inhibit metallo-β-lactamases (MBLs). Boronic acids and boronate esters, especially cyclic ones, can potently inhibit both SBLs and MBLs. Vaborbactam, a monocyclic boronate, is approved for clinical use, but its β-lactamase coverage is limited. Bicyclic boronates rapidly react with SBLs and MBLs forming stable enzyme–inhibitor complexes that mimic the common anionic high-energy tetrahedral intermediates in SBL/MBL catalysis, as revealed by crystallography. The ability of boronic acids to ‘morph’ between sp^2^ and sp^3^ hybridisation states may help enable potent inhibition. There is limited structure–activity relationship information on the (bi)cyclic boronate inhibitors compared to β-lactams, hence scope for creativity towards new boron-based β-lactamase inhibitors/antibacterials.

**Current Opinion in Chemical Biology** 2019, **50**:101–110

This review comes from a themed issue on **Next generation therapeutics**

Edited by **Yimon Aye** and **Paul J Hergenrother**

For a complete overview see the Issue and the Editorial

Available online 18th April 2019

**https://doi.org/10.1016/j.cbpa.2019.03.001**

1367-5931/© 2019 The Authors. Published by Elsevier Ltd. This is an open access article under the CC BY license (http://creativecommons.org/licenses/by/4.0/).

## Background

**Figure 1:**
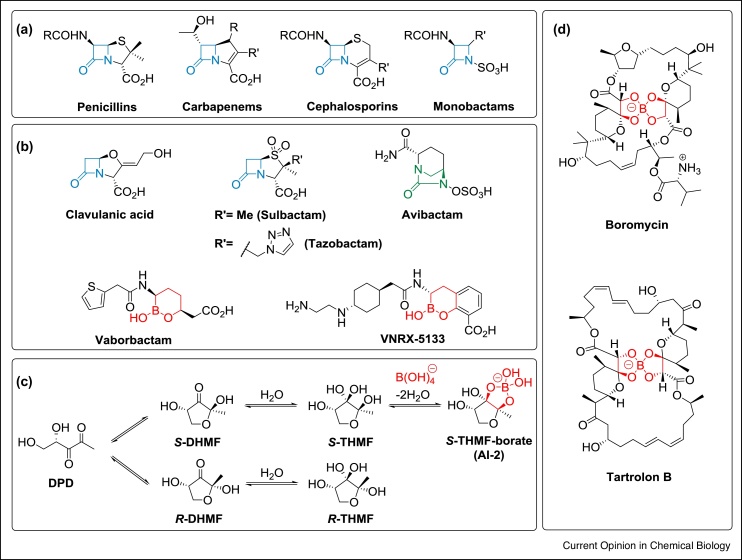
β-Lactam antibacterials, β-lactamase inhibitors and selected boron-containing natural products. **(a)** Major classes of β-lactam antibacterials; **(b)** clinically relevant SBL inhibitors (Clavulanic acid, Sulbactam, Tazobactam); the recently introduced non-β-lactam β-lactamase inhibitors Avibactam (a diazabicyclooctanone) and Vaborbactam (the first boron-containing β-lactamase inhibitor), and the candidate VNRX-5133 (Phase 3 compound). **(c)** Outline role of boron in quorum sensing in bacteria via borate complexation with (2*S*,4*S*)-2-methyl-2,3,3,4-tetrahydroxytetrahydrofuran (*S*-THMF) to produce the Autoinducer-2 (AI-2) which activates luminescence thus allowing bacteria to sense cell density [58,62]; **(d)** structures of two boron-containing natural products with antibacterial properties [63].

Following the clinical introduction of the penicillins in the 1940s, they and successive generations of β-lactam antibacterials, including cephalosporins, carbapenems and monobactams emerged as amongst the most important small molecule medicines (Figure 1a) [1]. The reasons for the reign of β-lactams as the predominant antibacterials are proposed to include their molecular suitability for inhibiting their bacterial targets in a safe and efficacious manner [2]. Political, financial and sociological factors also helped drive optimisation following the breakthrough discovery of the penicillins, aiming to expand the scope of β-lactam antimicrobial activity and to combat both pre-existing and emergent resistance [3].

**Figure 2:**
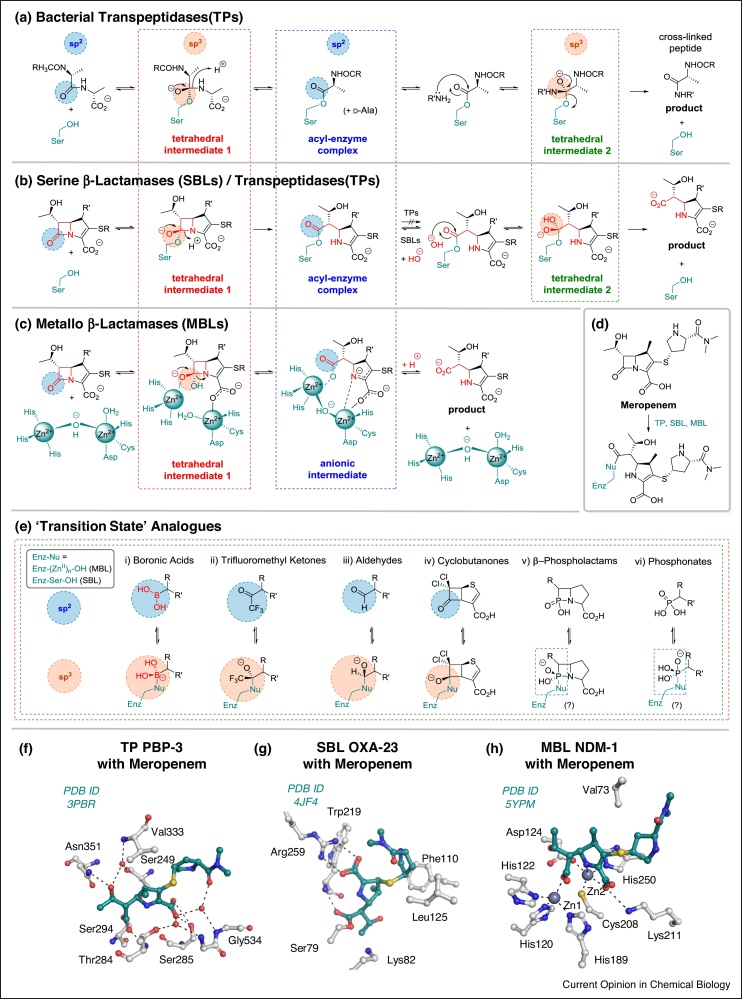
Modes of action of transpeptidases, serine-β-lactamases (SBLs) and metallo-β-lactamases (MBLs); potential transition state analogues and views from structures of reacted meropenem bound to representative transpeptidase, SBLs and MBLs. Outline mechanisms of **(a)** transpeptidase, **(b)** SBL and **(c)** MBL β-lactam (carbapenem) hydrolysis exemplified with **(d)** meropenem. Note the structural elements of the first anionic tetrahedral intermediate (in a red box) are common to SBLs and MBLs. **(e)** Selected examples of analogues of the first tetrahedral intermediate. Active site views from structures of meropenem in complex with **(f)** transpeptidase PBP-3 [64], **(g)** SBL OXA-23 [65] and **(h)** MBL NDM-1 [66]. Hydrogen-bond interactions are shown as dashed lines.

The targets of the β-lactams catalyse transpeptidase and carboxypeptidase reactions during bacterial cell wall biosynthesis in the periplasm. In the case of the essential transpeptidases this typically involves reaction of a donor peptide terminating in a d-Ala-d-Ala subunit to give an acyl–enzyme complex and d-alanine (Figure 2a). The acyl–enzyme complex then reacts with the *N*^ε^-amino group of a lysine on an adjacent peptide to give a crosslinked product, which is paramount for bacterial cell wall integrity [4]. The β-lactam ring of, for example the penicillins, in effect links the donor and acceptor units, enabling formation of an acyl–enzyme complex that is stable to cleavage by an acceptor peptide or hydrolysis. Tipper and Strominger proposed the penicillin structure mimics that of the d-Ala-d-Ala subunit [5]. At least with bicyclic β-lactams, it may be that their strained nature mimics a transpeptidase bound high-energy substrate conformation [6].

## The challenge of β-lactamases

Resistance in the form of β-lactamase catalysed hydrolysis to give inactive β-amino acids was apparent from the early stages of β-lactam development (Figure 2b) [7,8]. Subsequent widespread β-lactam use has correlated with the global emergence of resistance, including directly via (i) transpeptidase substitution or overproduction; (ii) efflux pump mediated expulsion; (iii) reduced β-lactam uptake; and (iv) β-lactamase catalysis [9]. To date β-lactamases appear the most important resistance mechanism with >1000 examples now identified. On the basis of sequence analysis, β-lactamases are grouped into four (Ambler) classes: A–D [10,11]. Classes A, C and D contain a nucleophilic serine at their active sites (serine-β-lactamases, SBLs) and may have evolved from transpeptidases; both SBL and transpeptidase catalysis proceeds via an acyl–enzyme complex [12,13]. Class B β-lactamases are mono-zinc, or more commonly, di-zinc ion dependent metallo-hydrolases (metallo-β-lactamases, MBLs) and likely have a different evolutionary origin (Figure 2c) [14].

SBLs have been combatted by modifying β-lactams to render them less SBL susceptible, for example, as manifested in some cephalosporins and carbapenems, as well as by developing specialised β-lactamase inhibitors for use in combination therapies [8]. The latter strategy resulted in Clavulanic acid as a class A, and Sulbactam and Tazobactam as class A and C inhibitors, respectively. All of these SBL inhibitors, although β-lactams, operate via formation of stable acyl–enzyme complexes (Figure 1b). The aforementioned three SBL inhibitors are used in combination with a β-lactam antibiotic (e.g. Augmentin: Clavulanate and Amoxicillin). However, they do not have broad spectrum activities against the extended spectrum SBLs (ESBLs) and class D β-lactamases, and do not inhibit MBLs [15]. Use of carbapenems possessing both antibacterial and SBL inhibition properties is increasingly compromised by evolving β-lactamase mediated resistance [1,15]. Increased observations of the class B MBLs are concerning, because they confer resistance to most β-lactam antibiotic classes, with the current exception of the monobactams [14,16].

The weaknesses of β-lactams to β-lactamases coupled with a desire to find alternatives to the β-lactam ring itself has motivated extensive medicinal chemistry efforts, extending back to the 1940s. Various alternative acylating agents have been explored, including, for example expanded lactam ring analogues of the β-lactams [6]. Clinically relevant success in this line finally came with the introduction of Avibactam, a diazabicyclooctane derivative with relatively broad spectrum activity against class A, C and some class D SBLs [6,17^••^]. Avibactam is used clinically in combination with the cephalosporin ceftazidime (Avycaz, Zavicefta) [18]. By contrast with most β-lactamases, which likely react irreversibly, Avibactam works by reversible covalent reaction with SBLs to give relatively hydrolytically stable acyl–enzyme type complexes [17^••^]. However, Avibactam is susceptible to some SBLs and does not inhibit MBLs [19]. There is thus interest in more drastic changes to the classical ‘acylating’ β-lactam transpeptidase/β-lactamase inhibitors.

## Transition state inhibitors of β-lactamases and transpeptidases

Both the formation and deacylation of acyl-enzyme complexes in transpeptidase and SBL catalysis are proposed to proceed via general acid and base catalysed reaction with a nucleophile to give a high-energy ‘tetrahedral’ (sp^3^ hybridised) intermediate [20] (Figure 2). This mechanistic analysis has inspired application of the ‘transition state/high-energy intermediate analogue’ approach to SBL/transpeptidase inhibition. As with proteases, various reversibly interacting electrophiles giving tetrahedral complexes have been explored, including phosphonates, trifluoromethyl ketones, aldehydes, β-phospholactams, cyclobutanones and others (Figure 2e) (see e.g. [21,22]). Of the classes investigated, the sp^2^ hybridised boronic acids/boronate esters show particular promise.

Interest in boron-based drugs arises substantially as a consequence of their nature as Lewis acids which are capable of reversibly reacting with a range of biologically relevant nucleophiles via their vacant p-orbital [23^•^]. Importantly, this property is preserved in aqueous media. However, there is potential for complexity, including via oligomerisation, in the chemistry of boronic acids/boronates. This property has been exploited in their use in dynamic combinatorial chemistry to identify enzyme inhibitors [24]. In water, boronic acids react to form a borate anion and a hydronium ion, hence their Brønsted acidity relates to their Lewis acidity [25,26]. The *p*K_a_ values of boronic acids vary substantially, ranging from 4 to 10, with boronate esters typically having lower *p*K_a_ values than the analogous boronic acids [25,26]. Thus, detailed investigations on boronic acid/boronate based inhibitors should take into account their precise nature in biologically relevant contexts, since this may impact on effective concentrations/potency. In the absence of such knowledge the potential for complexity should be a consideration.

Boronic acids are often proposed to be good transition state analogues for enzymes with amide/lactam/ester substrates, because in their sp^3^ hybridised form they can adopt a similar geometry to the proposed high-energy tetrahedral intermediates [27]. Consistent with this, the typical lengths of boron–carbon and boron–oxygen bonds, that is 1.57 and 1.40 Å respectively, are comparable to sp^3^ hybridised carbon–carbon and carbon–oxygen bonds, that is, 1.54 and 1.43 Å, respectively [28]. However, it should be noted that in catalysis the transition states are not necessarily fully tetrahedral. Further, at least in some cases, including β-lactamases, the sp^2^, rather than the sp^3^, hybridised form of an inhibitor may better mimic the substrate where the carbonyl group is sp^2^ hybridised. This ability of boron to ‘morph’ hybridisation states thus means boronate based inhibitors have potential to exploit highly optimised substrate binding modes (in their sp^2^ state) then ‘morph’ to form a ‘locked’ sp^3^ state that mimics binding of a high energy intermediate. This ability may be particular important in the case of enzymes that are highly evolved to efficiently bind their substrates, as the case for β-lactamases. The observation of fast on and slow off rates as observed for a β-lactamase in the case of a potent boronate inhibitor [29,30] is consistent with this proposal. A further element of complexity arises in the localisation of the charge in boronate inhibitor complexes. In some types of transition state analogues, for example trifluoromethyl ketones, which are also successful enzyme inhibitors [31] (Figure 2e), the oxygen localised nature of charge of the oxyanion tetrahedral intermediate is maintained in the analogous intermediate and inhibitor complexes. In the analogous boron species on the other hand, the negative charge is centred on the boron rather than the oxygen atom. However, despite this apparent difference, boronic acids can clearly be potent inhibitors of nucleophilic enzymes [23^•^].

**Figure 3:**
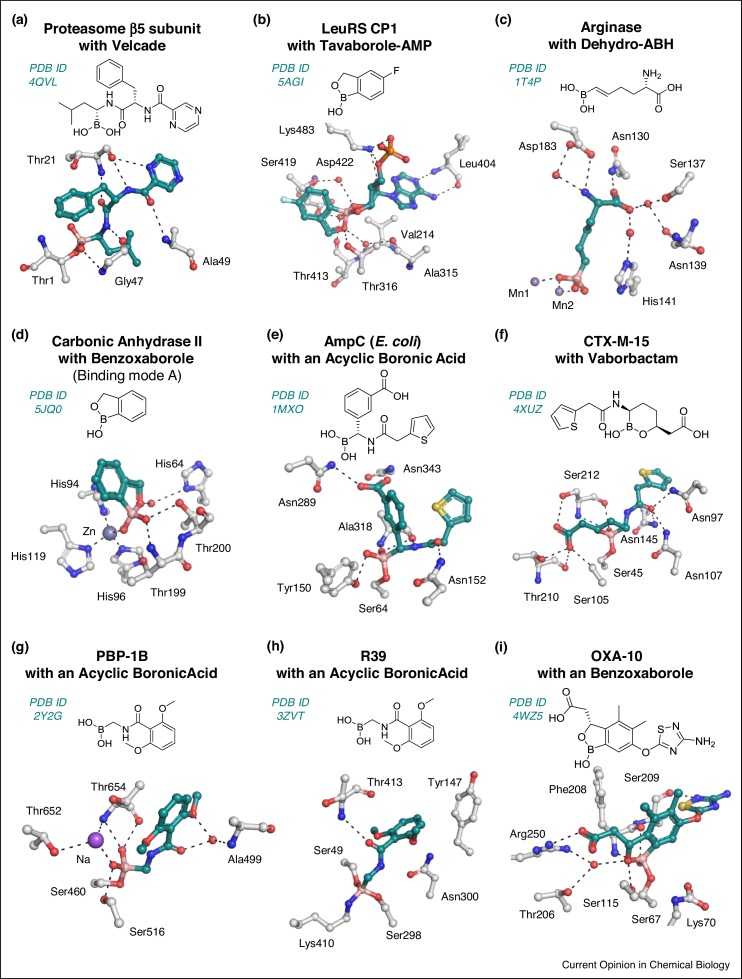
Structural analyses of boron-based enzyme inhibitors. Chemical structures of ‘boron’ inhibitors and active site views of **(a)** proteasome β5 subunit with Velcade [33]; **(b)** leucyl-tRNA synthetase (LeuRS) editing domain CP1 with Tavaborole-AMP [35]; **(c)** mammalian arginase with dehydro-2(*S*)-amino-6-boronohexanoic acid (dehydro-ABH) [67]; **(d)** human carbonic anhydrase II with benzoxaborole; note, two binding modes were observed. The major involves Zn(II) complexation via one of the exocyclic boronate oxygens (binding mode A, shown); in the minor (not shown) the inhibitor complexes via its endocyclic boronate oxygen and one of its exocyclic boronate oxygens [68]; **(e)** AmpC (*E. coli*) with an acyclic boronic acid [69]; **(f)** class A SBL CTX-M-15 with Vaborbactam [36]; **(g)** penicillin-binding protein 1b (PBP-1B) with an acyclic boronic acid [48]; **(h)** DD-transpeptidase (*Actinomadura* sp. R39) with an acyclic boronic acid showcasing an unusual tricovalent binding mode of the boronate [70]; **(i)** class D SBL OXA-10 with a benzoxaborole analogue [71].

Boronic acid transition state analogues have proven to be successful in the inhibition of proteases, notably in the case of the proteasome inhibition by Velcade (Bortezomib) and related drugs such as Ninlaro (Ixazomib) that are increasingly being used for treatment of multiple myeloma [32, 33, 34]. As revealed by crystallographic analysis, the boronic acid of Velcade reacts with the nucleophilic threonine residue of the proteasome to give a tetrahedral (sp^3^) boronate complex (Figure 3a) [33]. The pioneering example of the use of boron in drug discovery for proteasome inhibition has been followed by the introduction of bicyclic Tavaborole for the treatment of fungal nail infection (Figure 3b) [35].

Recent work has led to the clinical introduction of the first boronic acid based SBL inhibitor, Vaborbactam (Figure 1b), for use in combination with meropenem (Vabomere, Carbavance) [36,37]. Other boronic acids, especially bicyclic structures, are manifesting promise as (relatively) broad spectrum β-lactamase inhibitors, including some with MBL activity [38^••^,^39^,^29^]. Below we summarise work leading to these compounds and indicate why further work in the field of (bi)cyclic boron-based β-lactamase/transpeptidase inhibitors is desirable.

## Brief history of boron containing antimicrobials

The potential antibacterial properties of boron compounds were first reported in the 19th century [40]. Though boric acid and other simple boron-containing derivatives had long been known as enzyme inhibitors [41], an important subsequent observation came with the discovery in the late 1970s, that boric acid (B(OH)_3_) reversibly inhibits an SBL from *Bacillus cereus* [42]. This observation was followed by demonstration that the same SBL is inhibited by aryl-boronic acids that also inhibit serine proteases [8,43]. Subsequently, boronic acids were shown to inhibit representatives of class A, C and D SBLs, forming tetrahedral (sp^3^) boronate inhibitor complexes by reacting with the nucleophilic serine [44, 45, 46, 47]. This is also the case for Vaborbactam, as revealed by crystallography (Figure 3f) [36]. While the early boronic acid SBL inhibitors are apparently predominantly acyclic in solution, Vaborbactam, adopts a monocyclic structure, as observed at the active site of CTX-M-15 SBL (Figure 3f) [36].

**Figure 4:**
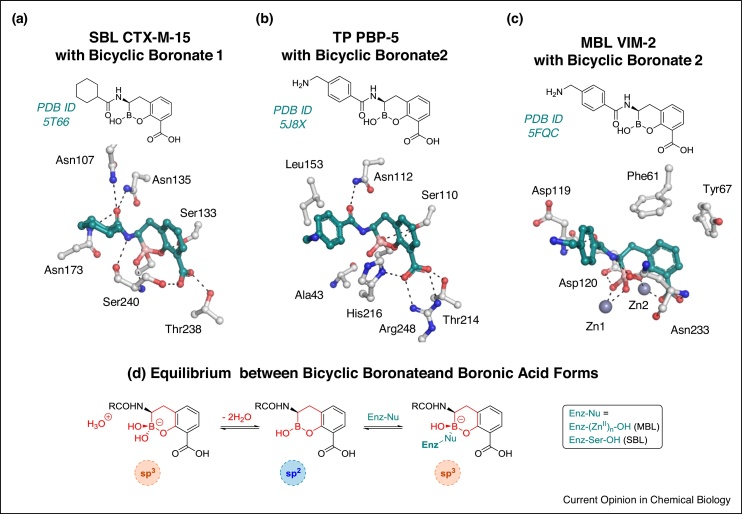
Structural analyses of bicyclic boronates with serine β-lactamases (SBLs), metallo-β-lactamases (MBLs) and transpeptidases. Views from crystal structures of Bicyclic Boronate 1 in complex with **(a)** SBL CTX-M-15 [30] and Bicyclic Boronate 2 complexed with **(b)** transpeptidase PBP-5 from *E. coli* [38^••^] and **(c)** MBL VIM-2 [38^••^]. **(d)** Current evidence implicates that sp^2^ boronic acid form may bind to the enzyme targets of the bicyclic boronate inhibitors.

Acyclic boronic acids have also been developed as transpeptidase inhibitors, as exemplified in work on methicillin-resistant *Staphylococcus aureus* (MRSA) acting compounds [48]. Multiple structures are reported for alkyl boronic acids bound to PBP-1B [48]. Subsequent work has defined boronic acid inhibitors that may more directly mimic the deacylation tetrahedral intermediate in class C SBLs [49^•^]. However, these compounds did not inhibit class A SBLs, transpeptidases, or d,d-carboxypeptidases (which catalyse d-Ala-d-Ala hydrolysis). It was proposed that these observations might reflect the slow deacylation of β-lactam derived acyl–enzyme complexes with transpeptidases/carboxypeptidases, which are proposed to be due to steric interactions, that is the same interactions may hinder formation of the analogous anionic boronate enzyme complexes [49^•^]. The observation that these boronic acids inhibit class C, but not class A SBLs was rationalised on the basis of active site differences. However, given that bicyclic boronates can potently inhibit representatives of class A and C SBLs (see below and Figure 4) [38^••^,^30^], the precise reasons for selectivity of some boronates for particular SBLs/transpeptidases requires further investigation [49^•^].

## Bicyclic boronic acids as inhibitors of both serine-β-lactamases and metallo-β-lactamases

Although SBLs and MBLs are structurally distinct, comparison of the proposed mechanisms for them (Figure 2) reveals that the first anionic tetrahedral intermediate is common to both reaction types. It was proposed that boronate esters capable of mimicking this intermediate may inhibit both SBLs and MBLs via formation of a tight complex [29,30]. It was envisaged that the presence of a bicyclic ring structure may conformationally constrain the boronate ester/borate anion, thus enhancing binding and enabling potent dual SBL/MBL inhibition [38^••^]. As described above it may also be that sp^2^ boronic acid/boronate ester species better mimic (binding of) β-lactam substrates than an sp^3^ borate ion [29]. Preliminary ^11^B NMR studies suggest that the sp^2^ form predominates at neutral pH, with the sp^3^ species predominating at a higher pH [29]. Thus, at least in principle, the sp^2^ form could be envisaged as the preferred form for binding to both SBLs and MBLs, with the sp^3^ species being preferred in terms of forming a stable enzyme–inhibitor complex (Figure 2, Figure 4).

MBL inhibition by boronic acids/boronate esters is precedented by work on other metallo-enzymes, including arginase and carbonic anhydrase [50,51]. Arginases contain two active site Mn(II) ions, which are proposed to be responsible for coordinating the substrate and a metal ion bridging ‘hydrolytic’ water/hydroxide ion, as observed in di Zn(II) ion utilising MBLs (Figure 3c,d) [8,38^••^]. When the boronate inhibitor binds to arginase, one of the boronate oxygens replaces the bridging water and a second boron bonded oxygen complexes one of the Mn(II) ions. Note that the work on benzoxaboroles and carbonic anhydrase reveals the potential for clinically relevant off-target interactions of boronic acid-/boronate ester-based SBL/MBL inhibitors.

## Mode of action and biophysical analysis of bicyclic boronate β-lactamase and transpeptidase inhibitors

Although the precise details of the kinetic mechanisms of binding of bicyclic boronates to β-lactamases remain to be established, results with representatives of classes A–D β-lactamases indicate the potential for potent broad spectrum inhibition, with the inhibitor concentration that decreases the rate to 50% in the specific assay (IC_50_) values often in the nanomolar range [38^••^,^39^,^29^,^30^]. SBLs for which potent inhibition was observed include the class A ESBLs, such as CTX-M-15, the class C AmpC from *Pseudomonas aeruginosa*, and a class D carbapenemase (OXA-48). Notably, a range of clinically relevant B1 subfamily MBLs, including VIM-2, IMP-1, NDM-1, and SPM-1 are also inhibited [38^••^,^39^,^29^,^30^]. Microbiology studies clearly support *in vivo* inhibition of SBLs/MBLs by bicyclic boronates. Cyclic boronates can potentiate the activity of penicillins, cephalosporins, monobactams, and carbapenems against Gram-negative clinical isolates expressing a variety of β-lactamases [30]. They also significantly improve the extent of meropenem susceptibility (minimum inhibitory concentration, MIC, ≤2 μg ml^−1^) against contemporary, clinical NDM and VIM MBL-positive *Enterobacteriaceae* [29].

In some cases, including the mono Zn(II) ion utilising B2 and some B3 subclass MBLs, (e.g. CphA and L1) inhibition by bicyclic boronates was not observed or was weak [38^••^,^39^]. While in the reported academic studies transpeptidase inhibition by bicyclic boronates is not always potent [38^••^], the patent literature implies that antibacterial boronates acting via transpeptidase inhibition are possible [52]. Thus, although results to date have demonstrated the potential for bicyclic boronates to inhibit a relatively broad range of SBLs/MBLs, understanding the underlying reasons (including those relating to Lewis and Brønsted acidity) for the relative lack of activity versus, for example, B2 MBLs as well as transpeptidases requires further work.

High-resolution crystallographic analyses reveal that the SBL, MBL, and transpeptidase (in the latter case, despite the lack of potent inhibition) binding modes of the core bicyclic ring systems of close relatives of VNRX-5133 [29,53^••^], are strikingly similar (Figure 4). In all cases the boron is clearly sp^3^ hybridised and in the case of all the SBLs/transpeptidases analysed, the nucleophilic serine is covalently bonded to the boron (Figure 4a,b). In the case of the VIM-2 and BcII MBLs, as revealed by crystallography, the boron is also sp^3^ hybridised [38^••^]. In the B1 subclass MBL bicyclic boronate complexes, binding of the ‘exocyclic’ boron bound oxygen/hydroxides involves both active site Zn(II) ions (Figure 4c). The *pro*-(*S*) exocyclic boron oxygen coordinates with Zn1, whereas the *pro*-(*R*) exocyclic oxygen bridges Zn1 and Asp120, in a manner analogous to that proposed for the hydroxide derived oxygen in the tetrahedral oxy-anion intermediate during β-lactam hydrolysis [38^••^]. The binding modes of the C-3 bicyclic boronate carboxylate also mimic those proposed for β-lactam substrate/inhibitor complexes (Figure 2, Figure 4). There is some variation in the observed conformations of the acetamido side chains, but such variation is common in β-lactamase/transpeptidase inhibition.

## So will boron-based inhibitors replace β-lactams?

The available evidence is that it is unlikely that boronate based inhibitors will quickly replace β-lactams as antibiotics. Indeed, to date, no stand-alone (cyclic)boronates targeting transpeptidases with similar potency and breadth of activity compared to clinically used β-lactam antibacterials have been developed for clinical application, though some boronate antibacterials are reported [54]. However, the reported results for both monocyclic (Vaborbactam [36]) and, especially, bicyclic boronates (VNRX-5133 and related compounds) clearly suggest that there is a potential for their use as broad spectrum β-lactamase inhibitors in conjunction with an appropriate β-lactam antibiotic [29,55,56]. Detailed studies on the precise mechanisms of action of boron-based inhibitors, including on exactly what sp^2^/sp^3^ species bind/don’t bind to their protein targets, may enable new generations of boron-based inhibitors. In appropriate cases, use of boron compounds to enhance activity or stabilise/destabilise specific protein folds are also of interest.

There will likely be challenges in optimising widespread clinical applications of boronate inhibitors at the relatively high doses required for antibacterial use, for example compared to typical cancer drugs. They will have to be optimised for use with a β-lactam partner; overcoming the propensity of boronic acids/boronates for pleiotropic reaction with nucleophiles may also be an issue. The efficacy of boron-containing drugs may also be affected by diet, and metabolism. For example, flavonoids, which are found in green tea, fruits, vegetables and wine, can react with boronic acids to form esters, which can affect drug properties, as in the case of Bortezomib [57]. Importantly, there is relatively limited structure–activity-relationship information on (bi)cyclic boronates, especially compared to the long history of such studies with β-lactams. Hence, there is considerable scope for optimisation and creativity in the design of new boronate-based β-lactamase inhibitors/antibacterials.

In principle the use of non-β-lactam/non-acylating chemotypes may have advantages with respect to β-lactamase mediated resistance, that is boronates should not be susceptible to (facile) β-lactamase hydrolysis. Boronates may also manifest different properties than β-lactams with respect to the induction of β-lactamases with initial observations looking promising in this regard [39,29]. It is conceivable that resistance may emerge less efficiently for cyclic boronates than for the naturally related β-lactams, which are common and for which resistance elements, including β-lactamases, have likely existed for hundreds of millions of years. However, it would be remarkable if efficient resistance to clinically used boronic acid antibacterials did not emerge. It will be interesting to observe if this is related to natural uses of boron, for example in quorum sensing [41,58], ionophoric polyketide macrolide antimicrobials (e.g. Boromycin and Tartolone B) [59,60], and in algae/plants (Figure 1c,d) [61]. Given the scant knowledge of boron compound modifying enzymes, it will be of particular biochemical interest to observe if unprecedented drug modifying reactions emerge in response to their clinical applications.

## Conflict of interest statement

Nothing declared.

## References and recommended reading

Papers of particular interest, published within the period of review, have been highlighted as:

• of special interest
•• of outstanding interest
